# Portability of the thiolation domain in recombinant pyoverdine non-ribosomal peptide synthetases

**DOI:** 10.1186/s12866-015-0496-3

**Published:** 2015-08-13

**Authors:** Mark J. Calcott, David F. Ackerley

**Affiliations:** School of Biological Sciences, Victoria University of Wellington, Wellington, New Zealand; Centre for Biodiscovery, Victoria University of Wellington, Wellington, New Zealand; Maurice Wilkins Centre for Molecular Biodiscovery, School of Biological Sciences, University of Auckland, Auckland, New Zealand

## Abstract

**Background:**

Non-ribosomal peptide synthetase (NRPS) enzymes govern the assembly of amino acids and related monomers into peptide-like natural products. A key goal of the field is to develop methods to effective recombine NRPS domains or modules, and thereby generate modified or entirely novel products. We previously showed that substitution of the condensation (C) and adenylation (A) domains in module 2 of the pyoverdine synthetase PvdD from *Pseudomonas aeruginosa* led to synthesis of modified pyoverdines in a minority of cases, but that more often the recombinant enzymes were non-functional. One possible explanation was that the majority of introduced C domains were unable to effectively communicate with the thiolation (T) domain immediately upstream, in the first module of PvdD.

**Results:**

To test this we first compared the effectiveness of C-A domain substitution relative to T-C-A domain substitution using three different paired sets of domains. Having previously demonstrated that the PvdD A/T domain interfaces are tolerant of domain substitution, we hypothesised that T-C-A domain substitution would lead to more functional recombinant enzymes, by maintaining native T/C domain interactions. Although we successfully generated two recombinant pyoverdines, having a serine or a *N5*-formyl-*N5*-hydroxyornithine residue in place of the terminal threonine of wild type pyoverdine, in neither case did the T-C-A domain substitution strategy lead to substantially higher product yield. To more comprehensively examine the abilities of non-native T domains to communicate effectively with the C domain of PvdD module 2 we then substituted the module 1 T domain with 18 different T domains sourced from other pyoverdine NRPS enzymes. In 15/18 cases the recombinant NRPS was functional, including 6/6 cases where the introduced T domain was located upstream of a C domain in its native context.

**Conclusions:**

Our data indicate that T domains are generally able to interact effectively with non-native C domains, contrasting with previous findings that they are not generally portable upstream of epimerisation (E) or thioesterase (TE) domains. This offers promise for NRPS recombination efforts, but also raises the possibility that some C domains are unable to efficiently accept non-native peptides at their donor site due to steric constraints or other limitations.

**Electronic supplementary material:**

The online version of this article (doi:10.1186/s12866-015-0496-3) contains supplementary material, which is available to authorized users.

## Background

**Fig. 1:**
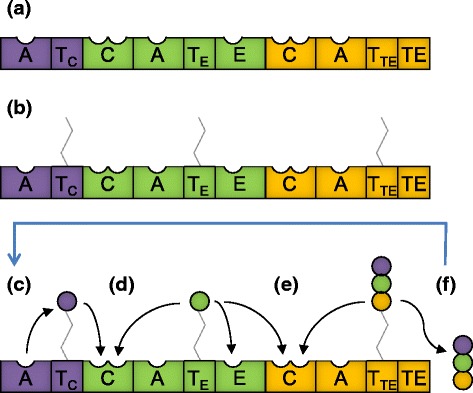
NRPS modular structure and mode of peptide synthesis. A = adenylation domain (substrate recognition and activation), T = thiolation domain (carrier protein), C = condensation domain (peptide bond formation), E = epimerisation domain (substrate racemisation), TE = thioesterase domain (product release). Most T domains are located immediately downstream of an A domain, but they can be located upstream of three different domain types. To indicate the downstream domain, the T domains in this figure are distinguished by an additional subscript label; e.g., the T domain of the initiation module is labelled T_C_ to indicate that it is located immediately upstream of a C domain. **a** Domain architecture of a three module NRPS containing an initiation module (core A-T domain structure; purple), an elongation module (core C-A-T domain structure, pictured here with an additional epimerisation domain; green) and a termination module (core C-A-T-TE domain structure; orange). (**b**) Biosynthesis occurs in an assembly line fashion, beginning with post-translational modification of each T domain by attachment of a 4’-phosphopantetheine (4’-pp) cofactor. The 4’-pp group functions as a flexible arm to coordinate the movement of substrates between catalytic sites. (**c**) The A domain within each module recognises and activates (via adenylation) a specific monomer, then attaches it to the 4′-pp group of the adjacent T domain. (**d** and **e**) In a stepwise manner, starting at the first module, each C domain catalyses peptide bond formation between the donor substrate attached to the upstream T domain and the acceptor substrate attached to the downstream T domain. Prior to this condensation reaction, each T domain may need to interact with additional tailoring domains, such as the E domain pictured in module 2, which can modify the substrate borne by each module. (**f**) When the peptide chain reaches the termination module, the product is released by a thioesterase (TE) domain via hydrolysis or intramolecular cyclisation. The cycle of synthesis may then repeat to generate many copies of the same peptide

Non-ribosomal peptide synthetases (NRPSs) produce a wide range of small peptide products of biotechnological interest. The peptide products can contain a highly diverse array of amino acids or related monomers, with over 600 different monomers having been detected in non-ribosomal peptides [1]. The diversity in monomers and peptide structure contributes to a wide range of bioactivities across all non-ribosomal peptides; for example, cytotoxic activities of the medically relevant peptides penicillin, daptomycin and cyclosporin A [2]. NRPSs synthesise peptides in an assembly line-like manner [3], with distinct modules governing the stepwise incorporation of specific monomers into the final product. The biosynthetic logic of this process is covered in-depth by many reviews [2, 4–6] and is summarised in Figure 1.

The modular nature of the NRPS assembly line offers promise for the creation of rationally modified peptides, or even combinatorial libraries of novel products, by recombining NRPS domains or modules at a genetic level. The primary substrate-specifying role of the A domain has made this domain a key target for NRPS recombination experiments. However, efforts to generate novel peptide products by modifying A domain substrate specificity, or by substituting in a non-synonymous A domain (that is, one which specifies an alternative monomer to the A domain it is replacing) have generally been unsuccessful [7, 8]. Numerous *in vitro* e.g., [9, 10] and *in vivo* [11, 12] studies have demonstrated that the C domain typically “proof-reads” the downstream acceptor substrate (Fig. 1d) in a manner that prevents incorporation of non-synonymous monomers into the peptide product. Structural studies have also indicated that disruption of the native C/A domain interface during A domain substitution may further impair the activity of recombinant NRPS modules [13]. Nonetheless, we found that five out of five synonymous A domain substitutions into PvdD (a bi-modular NRPS from *Pseudomonas aeruginosa* PAO1 that incorporates two L-threonine residues at the C-terminus of the peptide siderophore pyoverdine [14]) yielded recombinant enzymes that were highly active *in vivo*, whereas nine out of nine non-synonymous A domain substitutions were inactive [11, 12]. The 100 % success rate of the synonymous A domain substitutions suggests that disruption of native C/A or A/T domain interfaces was not a major factor restricting our ability to generate functional recombinant NRPS enzymes.

**Fig. 2:**
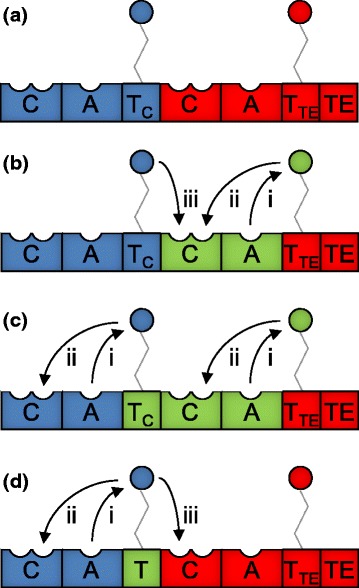
T domain interactions that are affected by substitution of alternative domains into PvdD. **a** Domain architecture of PvdD with the first module shown in blue and the second module shown in red. (**b-d**) Alternative domains substituted into PvdD are depicted in green: (**b**) C-A domain substitution; (**c**) T-C-A domain substitution; or (**d**) T domain substitution. In each panel, non-cognate (i.e., potentially disrupted) interactions between T domains and other domains are labelled according to the following scheme: (i) when an A domain passes a substrate to a non-cognate T domain; (ii) when a T domain passes a substrate to a non-cognate upstream C domain; or (iii) when a T domain passes a substrate to a non-cognate downstream C domain. The NRPS modules from which the C-A and T-C-A domains were sourced for substitution into PvdD are those labelled Ser1, Ser2 and fhOrn1 in Fig. 5

To overcome C domain proof-reading constraints, we next attempted to substitute cognate C-A domain partners into the second module of PvdD. This strategy successfully yielded two modified pyoverdine products, with L-serine or L-lysine in place of the terminal L-threonine present in the native pyoverdine. However, six of the eight C-A domain substituted constructs we generated were inactive, including two bearing threonine-specifying A domains that had previously been active when the A domain was substituted alone [12]. We proposed that one contributing factor to the inactivity of these six constructs might be an inability of the newly-introduced C domain to communicate effectively with the upstream T domain of the first PvdD module. Consistent with this proposal, other domain substitution studies have found that T domains upstream of C domains in their native context generally cannot function properly when placed upstream of E domains [15] or TE domains [16–19]. In two of the latter cases, it was further shown that T domain communication with the TE domain could readily be restored by point mutations introduced via error-prone PCR [18, 19]. Moreover, there is at least one example where dipeptides were successfully created *in vitro* via T-C-A domain substitution [16], a strategy that – unlike C-A domain substitutions – maintains native T/C domain interactions (Fig. 2a, b). Collectively, these studies suggest that T domain interactions with downstream domains may be particularly specialised, and that T domains may therefore have limited portability during NRPS recombination experiments. To highlight this potential specialisation and distinguish different subtypes of T domain on the basis of their downstream partner, when relevant in this manuscript T domains are labelled with the type of domain immediately downstream noted in subscript (e.g., Figure 1, Fig. 2).

**Fig. 3:**
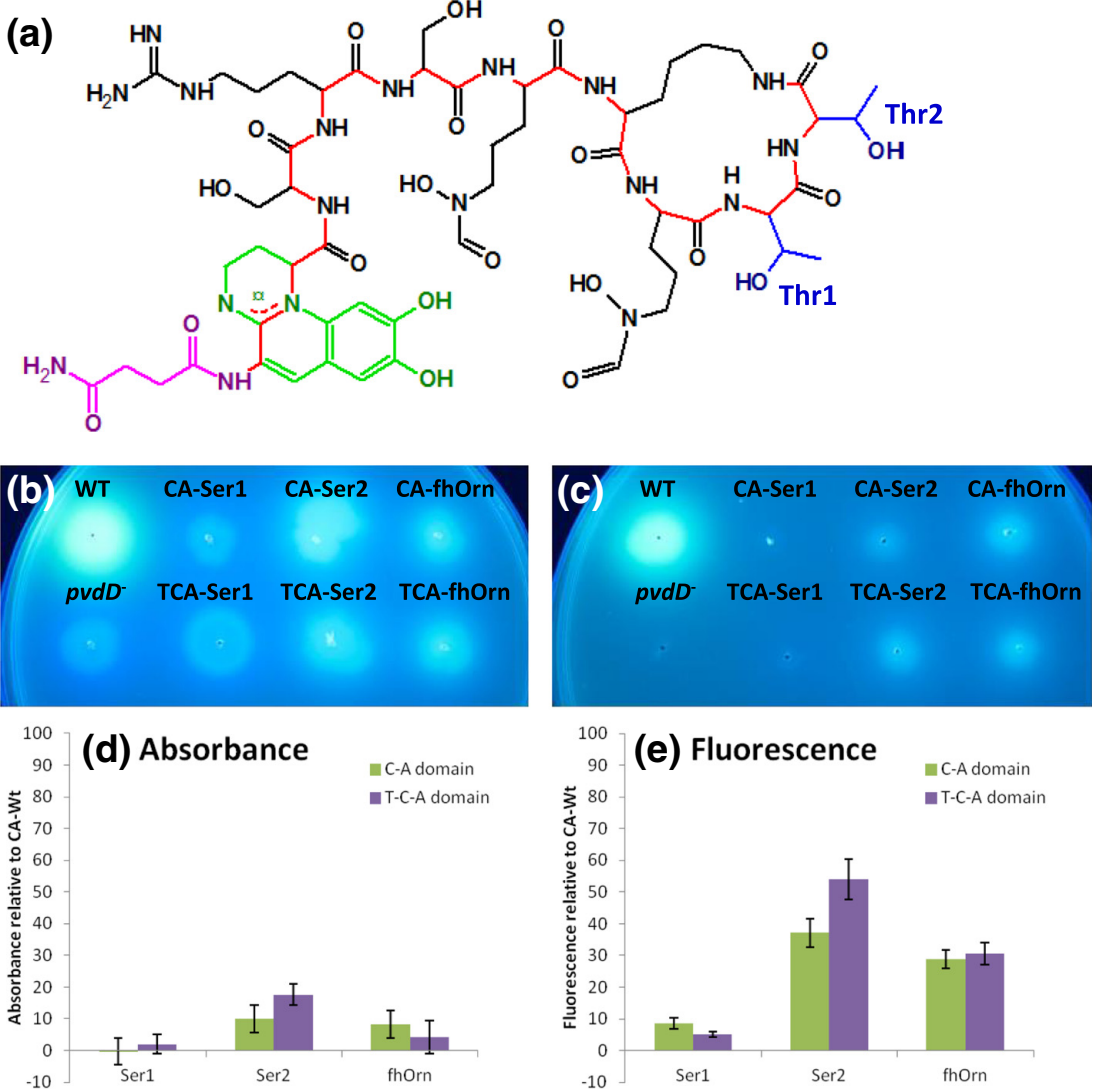
Growth of C-A *versus* T-C-A domain substitution strains. **a** Structure of pyoverdine from *P. aeruginosa* PAO1, with the succinimide group highlighted in pink, the chromophore in green, the peptide backbone in red, and the side chains of the threonine residues incorporated by PvdD in blue. The threonine residues incorporated by modules 1 and 2 of PvdD are labelled Thr1 and Thr2, respectively. (**b**) and (**c**): Growth of C-A and T-C-A substitution strains on solid King’s B media, either (**b**) without or (**c**) with addition of 200 μg.ml^−1^ EDDHA. Plates were inoculated with the restriction site control (WT), domain substitution strains, and the *pvdD* deletion mutant (*pvdD* ^*−*^), then incubated for 24 h at 37 °C. Photographs were taken under UV light. (**d**) and (**e**): Pyoverdine production in liquid media from T-C-A *versus* C-A domain substitution strains. Values are expressed as a percentage relative to (**d**) the absorbance (400 nm); or (**e**) the fluorescence (ex. 400 nm/ em. 440 nm) measured for the CAThr-WT strain, having first been zeroed against the absorbance/fluorescence of the *pvdD* deletion mutant. Data are the mean of 6 independent replicates, and error bars indicate 1 standard deviation

To evaluate the general portability of T domains, we again turned to the pyoverdine synthetase PvdD as a model NRPS enzyme for domain substitution experiments. This system offers the key advantages that pyoverdine is easily detectable *in vivo* due to the characteristic absorbance and fluorescence of its chromophore [20, 21] (Fig. 3a), that a conditional selection for pyoverdine synthesis can be imposed by growing the *P. aeruginosa* host in an iron-restricted environment, and that our previous work has identified recombination sites between the T-C, C-A, and A-T domain boundaries that permit effective substitution of alternative domains into PvdD [11, 12]. Furthermore, we have access in our laboratory to four different genome-sequenced strains of fluorescent pseudomonads that each synthesise a different pyoverdine peptide, providing a range of evolutionarily related modules for domain substitution. We used these resources to first conduct a pilot study of whether T-C-A domain substitution into PvdD might prove superior to C-A substitution as a means of generating novel pyoverdine peptides, and then performed an extensive survey of the ability of different types of T domain to communicate effectively with non-native C domains.

## Results and discussion

### Comparison of T-C-A and C-A domain substitution variants

To substitute alternative domains into PvdD we used an integrating vector bearing a copy of the *pvdD* gene that had restriction sites introduced, to enable replacement of either the native C-A domains of the second NRPS module, or the native T-C-A domains spanning the first and second modules (Fig. 2a, b). Three T-C-A domain substitution constructs were created and compared to equivalent constructs in which only the corresponding C-A domain substitutions had been made. Two of the C-A domain pairings encode a serine residue in their native context, and PvdD C-A substitution constructs bearing each of these alternative domain pairings had been analysed in our previous study [12]; in that work one (here called CA-Ser1) was non-functional and did not yield any detectable pigment, whereas the other (CA-Ser2) was found to incorporate a serine residue into pyoverdine. The third C-A domain pairing (used to make construct CA-fhOrn) was from a *N5*-formyl-*N5*-hydroxyornithine activating pyoverdine synthetase module from *P. fluorescens* SBW25, which had not previously been tested in domain substitution studies. The corresponding T-C-A domain substitution constructs were named TCA-Ser1, TCA-Ser2 and TCA-fhOrn.

Each C-A and T-C-A domain substitution construct was transformed into a *pvdD* mutant strain of *P. aeruginosa* PAO1, and analysed for production of pyoverdine (structure depicted in Fig. 3a) alongside a restriction site positive control strain (CA-Wt) and an empty plasmid negative control strain (*pvdD*^*−*^). As a preliminary test for pyoverdine production, each strain was spotted onto iron-limiting King’s B agar plates in either the absence (Fig. 3b) or presence (Fig. 3c) of 200 μg.ml^−1^ of ethylenediamine-N,N’-bis(2-hydroxyphenylacetic acid) (EDDHA; an iron-chelating agent that prevents passive uptake of iron). Of the C-A domain substitution strains, those bearing constructs CA-Ser2 and CA-fhOrn were fluorescent under UV and able to grow in the presence of EDDHA, whereas the strain containing construct CA-Ser1 was neither fluorescent nor able to out-compete EDDHA for iron. The T-C-A domain substitution strains showed similar results to the corresponding C-A domain substitution strains; that is, the strains bearing domain substitution constructs TCA-Ser2 and TCA-fhOrn were both fluorescent and able to grow in the presence of EDDHA, whereas the strain containing construct TCA-Ser1 was not.

To more accurately quantify the levels of pyoverdine production, the absorbance (Fig. 3d) and fluorescence (Fig. 3e) for each T-C-A and C-A domain substitution strain was measured from the supernatant of 24 h cultures. As previously demonstrated, relative absorbance is a less sensitive but more linear measure of the relative amounts of pyoverdine present, whereas fluorescence is better able to detect low levels of pyoverdine [12]. By either measure, there did not appear to be a substantial difference between the C-A and T-C-A domain substitution strategies in terms of the overall yield of pigment produced.

**Fig. 4:**
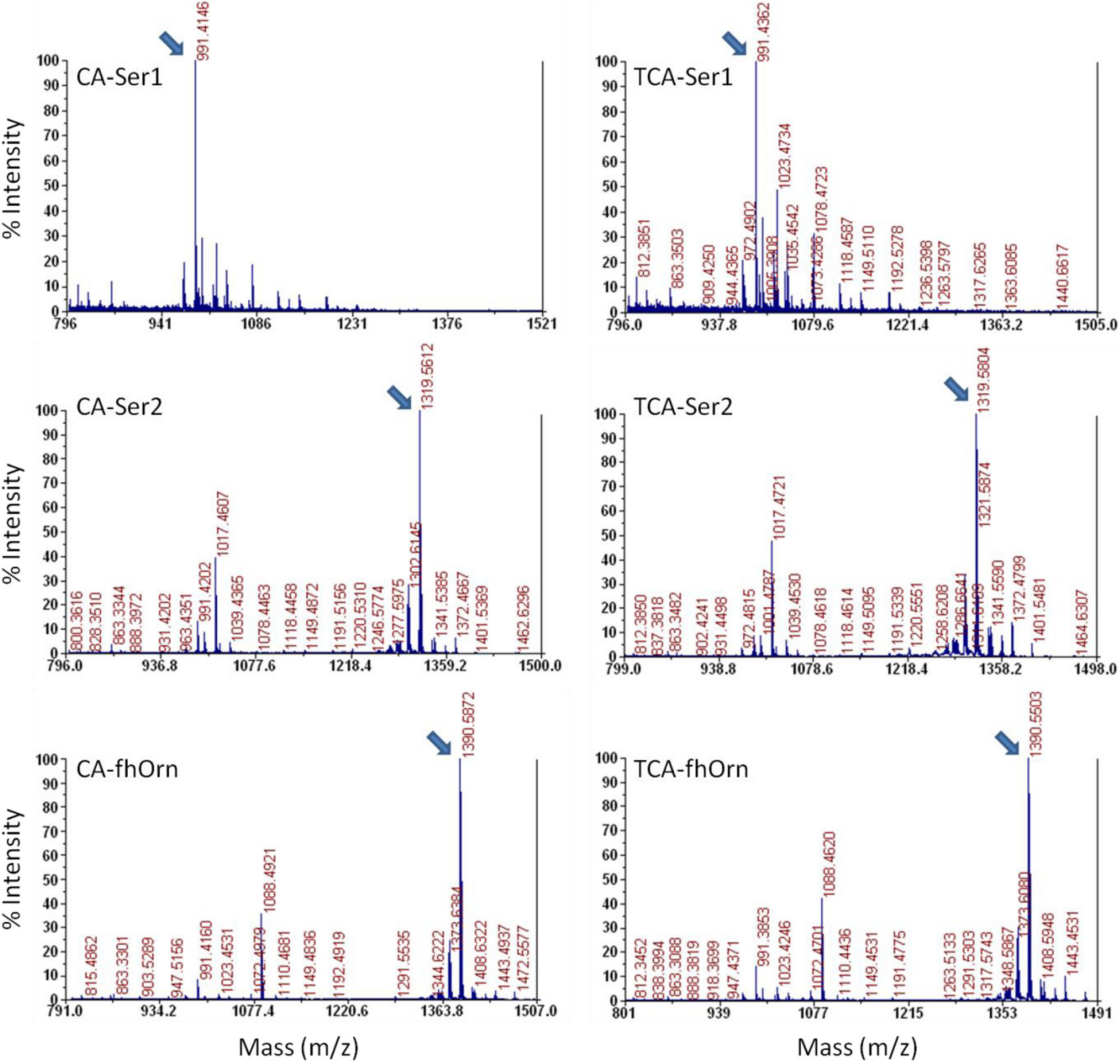
Mass spectra obtained from the supernatant of each of the C-A and T-C-A domain substitution strains. A product of *m/z* 991.5 was detected for CA-Ser1 and TCA-Ser1, which is consistent with the truncated pyoverdine species known to be produced by CA-Ser-1 [12]. The strains CA-Ser2/TCA-Ser2 and CA-fhOrn/TCA-fhOrn were found to produce products of *m/z* 1319.6 and 1390.6, respectively. These products were consistent with pyoverdine containing a terminal serine residue and a terminal *N5*-formyl-*N5*-hydroxyornithine residue, respectively

The identity of the pyoverdine produced by each strain was then confirmed by MALDI-TOF mass spectrometry (MS) (Fig. 4). The strains CA-Ser1 and TCA-Ser1 each produced low levels of a truncated pyoverdine, a species that had previously been shown to be released by strain CA-Ser1 [12]. The other strains produced full-length pyoverdines consistent with the known substrate specificity of the substituted A domain, that is CA-Ser2 and TCA-Ser2 produced pyoverdine derived from a peptide containing a C-terminal serine residue and CA-fhOrn and TCA-fhOrn produced an entirely new pyoverdine peptide that contained a fhOrn residue in place of the terminal threonine of wild type pyoverdine. In both cases, the mass of the primary product indicated that the final pyoverdine species was still able to cyclise via peptide bond formation between the terminal carboxyl of the substituted amino acid and the amino group bound to the ε-carbon of an internal lysine residue.

### Analysis of T domain portability

The lack of evidence for T-C-A domain substitution being a fundamentally superior strategy to C-A domain substitution for generating functional recombinant PvdD enzymes, together with a relative paucity of suitable contiguous T-C-A domain combinations available for substitution, and technical difficulties in amplifying and cloning such large DNA inserts, dissuaded us from building additional T-C-A domain substitution constructs. Instead, to focus more specifically on whether transplanted T domains might have difficulty in communicating effectively with non-native C domains, a series of T domain substitutions were made by replacing the first T domain of PvdD according to the substitution scheme represented in Fig. 2c.

When considered in the context of altered domain interactions, T domain substitutions are fundamentally similar to C-A domain substitutions; that is, both establish a scenario in which there is perturbation of (i) native A/T domain interactions; (ii) upstream C and downstream T domain interactions; and (iii) upstream T and downstream C domain interactions (Fig. 2a, 2c). As we previously found five out of five synonymous A domain substitutions into PvdD to be active [11, 12], it can be inferred that A domains are readily able to communicate with non-native T domains. We therefore reasoned that our proposed T domain substitution strategy would shed light on whether non-native T and C domains can also communicate effectively.

**Fig. 5:**
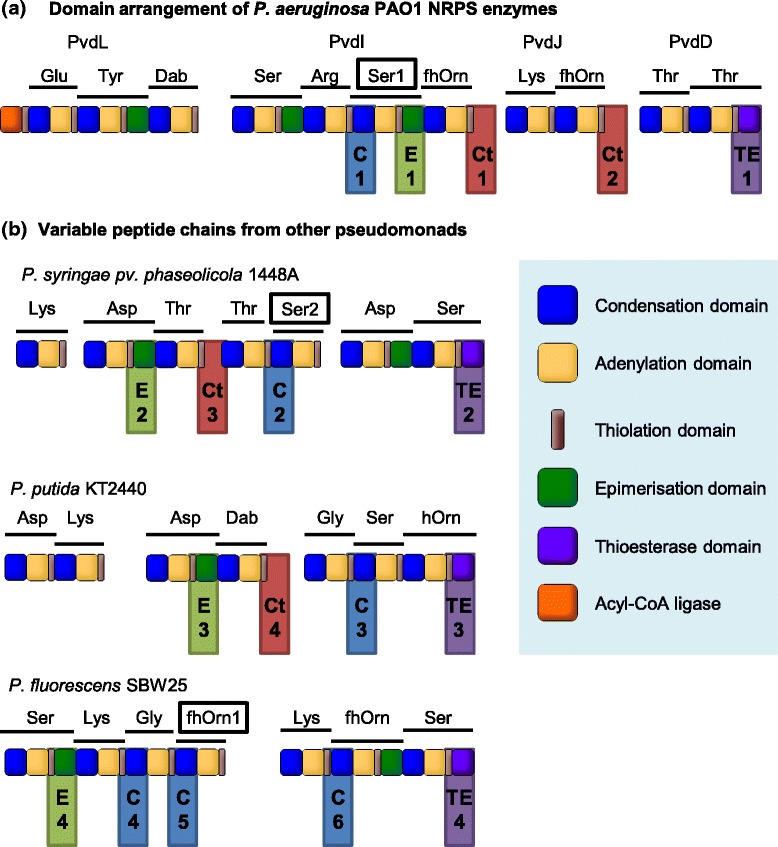
Location of T domains within *Pseudomonas* pyoverdine NRPS gene clusters that were used to substitute the T1 domain of *pvdD*. **a** Domain architecture of the four NRPS genes involved in *P. aeruginosa* PAO1 pyoverdine synthesis. (**b**) Domain architecture of genes involved in the synthesis of pyoverdine variable peptide chains from three other *Pseudomonas* strains. Location and numbering of the six T_C_ (blue), four T_Ct_ (red), four T_E_ (green), and four T_TE_ (purple) domains used for domain substitution are indicated by the colour-coded shaded boxes. Standard abbreviations are used to identify the amino acid substrate incorporated by each module, with the following additions: Dab, 2,4-diaminobutyric acid; fhOrn, L-*N5*-formyl-*N5*-hydroxyornithine; hOrn, *N5*-hydroxyornithine. Within this figure the three modules from which domains were sourced for C-A and T-C-A domain substitution are identified by rectangles around the name of the substrate activated by that module as well as addition of numbers after that substrate name, to correspond with the nomenclature used in the C-A and T-C-A domain substitutions

Eighteen T domain substitution constructs were created, using a variety of T domains sourced from the *P. aeruginosa* PAO1, *P. syringae* 1448a, *P. putida* KT2440 and *P. fluorescens* SBW25 pyoverdine NRPS gene clusters (Fig. 5) (T domain exchange boundaries and amino acid identities shared with the substituted PvdD T domain, as well as NRPS accession numbers, are summarised in Additional file 1: Table S1). T domains from each subtype were selected to test whether there were any clear differences in the ability of T_C_, T_E_ or T_TE_ domains to function effectively when transplanted immediately upstream of a C domain. An additional nomenclature, T_Ct_, was introduced to distinguish T_C_ domains that act *in trans* with a downstream C domain in their native context (Fig. 5, red highlighted T domains) *versus* the more common T_C_ domains that act *in cis* with a downstream C domain (Fig. 5, blue highlighted T domains). This distinction was made to allow for the possibility that T_Ct_ domains may carry additional recognition elements, in addition to short communication domains, that allow them to associate with the correct downstream C domain [22, 23]. In total, six T_C_ domains and four of each of T_Ct_, T_E_ and T_TE_ domains were substituted into the first module of PvdD, giving rise to T domain substitution strains C1-C6, Ct1-Ct4, E1-E4, and TE1-TE4 as explained in Fig. 5. The substituted regions ranged from 34.6 % to 61.5 % shared identity with the corresponding sequence from PvdD (Additional file 1: Table S1).

**Fig. 6:**
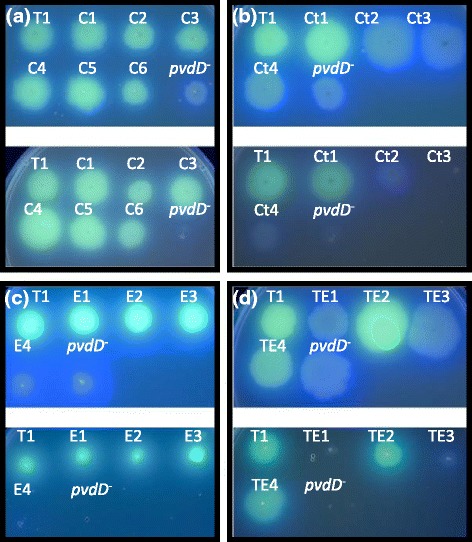
Growth of T1 domain substitution strains on solid media. Substitutions are grouped as **a** T_C_ domain substitutions, (**b**) T_Ct_ domain substitutions, (**c**) T_E_ domain substitutions or (**d**) T_TE_ domain substitutions. For each type of substitution, the top panel is for growth on KB media and the bottom figure is for growth on KB media supplemented with EDDHA. T1 refers to the restriction site control of the native T_C_ domain ligated back into pST1; and *pvdD* ^*−*^ is the *pvdD* deletion mutant negative control. Plates were incubated for 24 h at 37 °C, and photographs were taken under UV light

The 18 T domain substitution strains, together with the empty plasmid negative control strain (*pvdD*^*−*^) and the restriction site positive control (T1), were spotted onto KB agar plates both with and without EDDHA (Fig. 6). Each of the T_C_ domain substitution strains (C1-C6) were fluorescent at levels indistinguishable from control strain T1 and were all highly viable in the presence of EDDHA (Fig. 6a). In contrast, the other T domain substitution strains varied greatly in their fluorescence and ability to grow in the presence of EDDHA, with at least one strain for each T domain subtype being largely non-fluorescent on KB agar and greatly impaired in growth in the presence of EDDHA (Fig. 6, panels b–d).

**Fig. 7:**
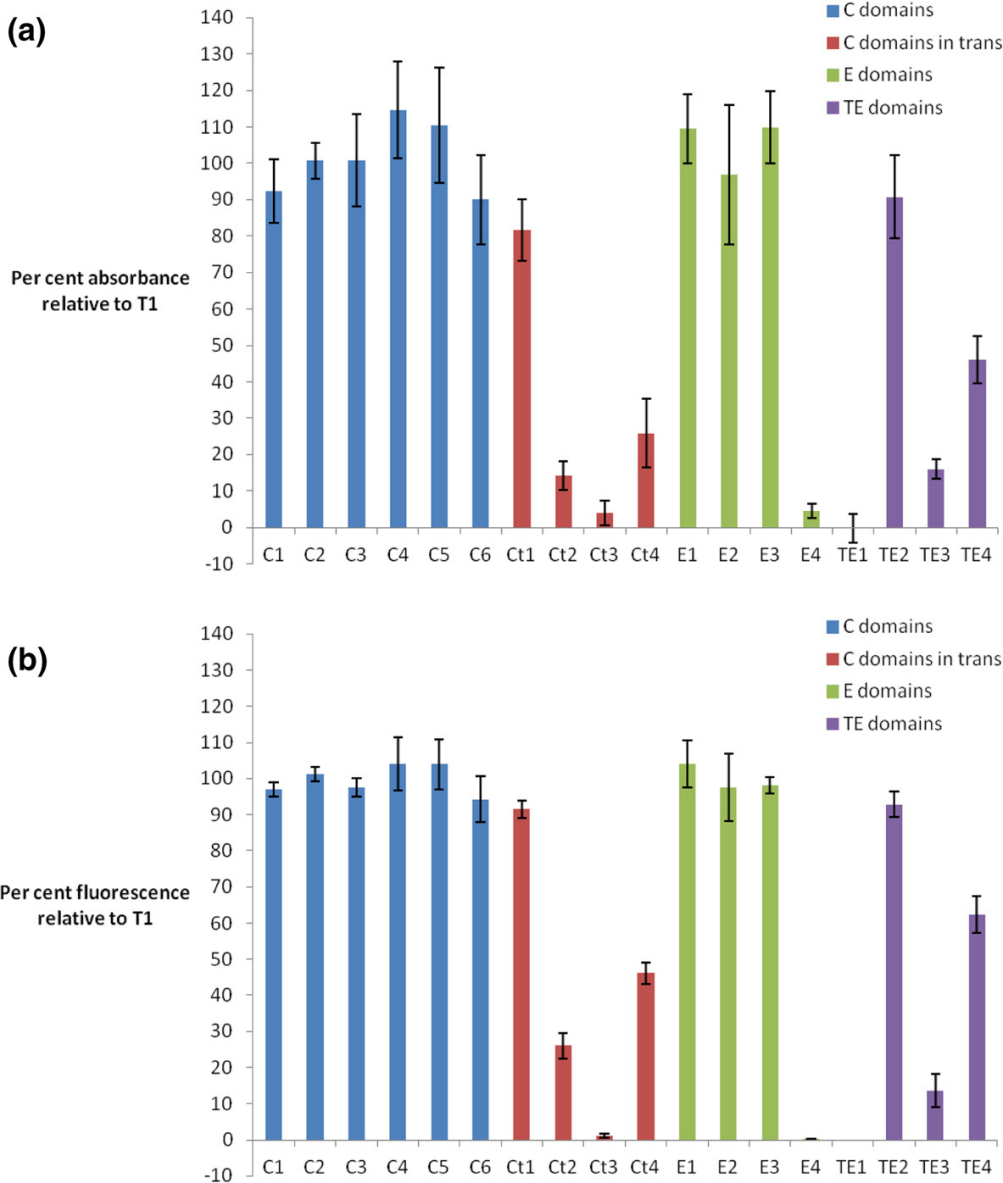
Pyoverdine production from *pvdD* T1 domain substitution strains grown in liquid media. Values are expressed as a percentage relative to the **a** absorbance (400 nm) or (**b**) fluorescence (ex. 400 nm/em. 440 nm) measured for the T1 restriction control strain, having first been zeroed against the absorbance/fluorescence of the *pvdD* ^*-*^ deletion mutant negative control strain. Data are the mean of 6 independent replicates, and error bars indicate 1 standard deviation

When grown in liquid media, the levels of absorbance (Fig. 7a) and fluorescence (Fig. 7b) of supernatant from cultures of each of the T domain substitution strains were consistent with the qualitative findings on solid media (Fig. 6). Whereas strains C1-C6 were all found to produce high levels of pyoverdine, one of each of the T_Ct_, T_E_ and T_TE_ substitution strains appeared to make no pyoverdine at all (Ct3, E4, TE1), while others (Ct2, Ct4, TE3, TE4) were severely impaired in their level of pyoverdine production. However, the remaining T_Ct_, T_E_ and T_TE_ substitution strains produced near wild type levels of pyoverdine.

**Fig. 8:**
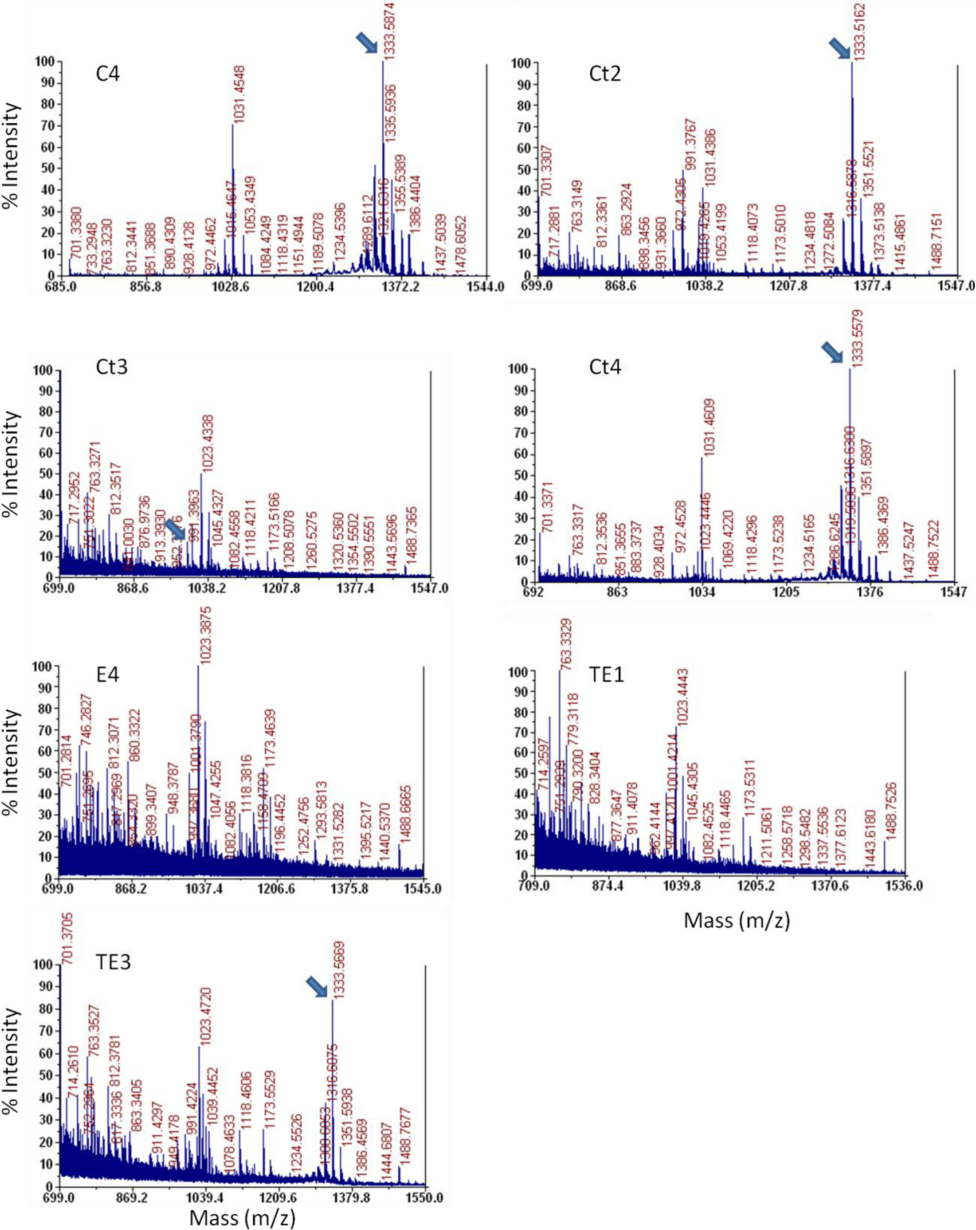
Mass spectra obtained from the supernatant of each of the T1 domain substitution strains. Arrows indicate peaks at 991.5 or 1333.6 m/z, corresponding to the masses of the previously detected truncated and wild-type pyoverdine species, respectively

MALDI-TOF MS was used to analyse the supernatants from cultures that exhibited limited or no apparent pyoverdine production. For the apparent non-producing strains E4 and TE1, no pyoverdine-like products could be detected. However, the other apparent non-producing strain Ct3 was detected to synthesise the same truncated 991.5 m/z product (Fig. 8a) that had been detected in culture supernatants from the non-functional strains CA-Ser1 and TCA-Ser1 (Fig. 4). The remaining T domain substitution strains were all found to produce wild type pyoverdine (Fig. 8).

Thus, only 3 out of the 18 T domain substitution strains were completely unable to synthesise pyoverdine, none of these being strains in which the native T_c_ domain of PvdD had been substituted by another T_c_ domain. These data suggest that T domains transplanted upstream of a non-native C domain generally retain functionality, contrasting with earlier studies in which T_C_ domains placed upstream of non-native E [15] or TE [16–19] domains were severely impaired or inactive. Our data also confirm that the boundaries we selected between the PvdD A-T and T-C domains were appropriate recombination sites for T domain substitution. This is an important point; in one other previous study it was found that the T_TE_ domain from the single module indigoidine synthetase BpsA from *Streptomyces lavendulae* was unable to substitute for the T_TE_ domain of the closely related single module indigoidine synthetase IndC from *Photorhabdus luminescens* until optimal recombination sites were identified [24].

## Conclusions

In previous work we found that a majority of C-A domain substitutions in the second module of PvdD yielded inactive recombinant enzymes [12]. We hypothesised that this might stem from the T domain of the first PvdD module being unable to communicate effectively with the newly-introduced C domain. If this hypothesis were true, then T-C-A domain substitutions would be expected to prove superior to C-A domain substitutions as a means of generating novel non-ribosomal peptide products. Although T-C-A domain substitution has previously been used to successfully create a bi-modular recombinant NRPS that was functional *in vitro* [16], the authors did not construct the equivalent C-A domain substitution construct to compare relative activities. Here, while we were able to use T-C-A domain substitution to successfully generate an entirely new pyoverdine species that had fhOrn at the C-terminus, and as an alternative means of producing a serine-substituted pyoverdine we had previously generated via C-A domain substitution [12], we did not observe substantial differences between the T-C-A substitution and C-A domain substitution strategies in terms of product yield.

We also found that T domains were generally tolerant of being relocated immediately upstream of the C domain of the terminal PvdD module, particularly if the introduced T domain had been located immediately upstream of a C domain in its native context. That is, within their new environment they were generally capable of interacting effectively with non-native C and A domains upstream, and a non-native C domain downstream. This capability appeared to be independent of the amino acid identity shared between the introduced T domains and the PvdD T domain that they were replacing. For example, the T_Ct_, T_E_ and T_TE_ domains that shared the highest sequence identity with the PvdD T domain were actually the least active when substituted into PvdD (Additional file 1: Table S1). We consider this general portability of T domains to locations upstream of C domains to be quite remarkable, in light of the extreme conformational and functional demands that are placed on T domains [13, 25–27], as well as previous studies indicating that T_C_ domains are not generally portable upstream of E or TE domains [15–19]. From a biotechnology perspective it is a promising finding, as it suggests that T domain functional incompatibility is unlikely to be a major driver of recombinant NRPS inactivity following C-A or T-C-A domain substitution.

Collectively, our data suggest that the low success rates of our previous C-A domain substitutions into module 2 of PvdD [12] were not due to inability of the newly introduced C domains to communicate effectively with the PvdD module 1 T domain. Rather, we propose that the loss of function may have been due to inability of the introduced C domain to receive the large incoming pyoverdine peptide at its donor site, possibly due to steric constraints. The original work of Belshaw et al., which demonstrated no side-chain amino acid specificity at the donor site of C domains, used only single amino acids as donor substrates [9]. In contrast, later work by Stein et al. identified specificity within the C domain donor site for some dipeptides [28]. Similarly, Clugston et al. [29] observed stereo specificity at the C domain donor site towards a tetrapeptide, but not towards the single amino acid substrate. The studies by Stein et al. [28] and Clugston et al. [29] strongly suggest that the size and composition of the incoming donor peptide can influence activity. Thus, steric constraints at the donor site position might explain the variable success rate of C domain substitutions, with some C domains being more tolerant of the new peptide chain. Ultimately, this would mean that both A and C-A domain substitution strategies are impeded by C domain limitations – the former by acceptor site selectivity, and the latter by donor site constraints. Overall, a greater understanding of C domain specificity is needed as it may aid in future efforts to perform functional NRPS domain substitutions.

## Methods

### Bacterial strains and growth conditions

*E. coli* DH5α, *P. aeruginosa* PAO1 and *P. putida* KT2440 were sourced from existing Ackerley lab stocks. *P. syringae* pv. phaseolicola 1448A was generously provided by Prof. John Mansfield (Imperial College, London), and *P. fluorescens* SBW25 by Prof. Paul Rainey (Massey University, Auckland, NZ). All strains were grown in LB media with shaking at 200 rpm, at 28 °C for *P. syringae* pv. phaseolicola 1448A and 37 °C for other strains. For maintenance of plasmids, tetracycline was added to a final concentration of 15 μg/ml for *E. coli* and 100 μg/ml for *P. aeruginosa* PAO1. Where necessary, to prevent passive uptake of iron, EDDHA was added to a final concentration of 200 μg.ml^−1^.

### General DNA methodology

All PCR primers and plasmids used in this study are shown in Additional file 2: Tables S2 and S3, respectively. Primers were designed to amplify pyoverdine NRPS domains from the genomes of fluorescent pseudomonads based on previous annotation by Owen and Ackerley [30] (*P. syringae* 1448a), Moon et al. [31] (*P. fluorescens* SBW25), and Ravel and Cornelis [32] (*P. putida* KT2440) as well as the NRPS analysis tools available online (http://nrps.igs.umaryland.edu/nrps/; [33]). PCR reactions used Phusion™ DNA polymerase (Finnzymes; Espoo, Finland). Plasmids created in this work were sequence verified by Macrogen Inc. (Seoul, South Korea).

### C-A and T-C-A domain substitution

C-A and T-C-A domain substitution plasmids were constructed from pSW196 [34] in *E. coli* DH5α. C-A domain constructs were created as previously described [8], with the primers CA-fhOrn_Fwd and CA-fhOrn_Rev used to amplify the new C-A domain pairing. To generate the T-C-A domain substitution plasmid pTCA, the C-A domains from the first module of *pvdD* were PCR amplified using primers CATfwd and CA-Wt_Rev and ligated into pSW196 using the *Not*I and *Sac*I restriction sites. The reverse primer introduced a *Not*I site in exactly the same location that was used for A domain substitutions previously [11]. Then, the T-TE domains from the second module of *pvdD* were introduced into the construct as previously described for the C-A domain substitution plasmid [12]. Target T-C-A domains were then PCR amplified using the corresponding primer sets listed in Additional file 2: Table S2, and introduced into the substitution plasmids via *Not*I restriction digest and ligation. Correct orientation of the introduced T-C-A domain amplicons was confirmed by sequencing. The resulting plasmids were then transformed into *P. aeruginosa* PAO1 for single-copy integration at the *attB* locus [35].

### T domain substitution

The domain substitution plasmid pST1 was derived from the plasmid pTCA. The second module of *pvdD* was amplified using the primers CATTE_Fwd and TTeRev. Next the PCR product and pTCA vector were each digested with *Not*I and *Sac*I, and the PCR product was ligated into pTCA. This replaced the T-Te domains of pTCA with the PCR amplified second module of *pvdD*. The primer CATTE_Fwd had added *Not*I and *Spe*I restriction sites immediately upstream of the C domain of the second module of *pvdD*, which allowed the subsequent insertion of T domains into these restriction sites. T domains for substitution into pST1 were amplified from the genomes of different fluorescent pseudomonads as indicated in Fig. 5 and Additional file 1: Table S1, using the primers listed in Additional file 2: Table S2 (primers being named according to the T domain they amplify, with F indicating a forward primer, and R a reverse primer; e.g., the primers for creating strain C1 are named C1F and C1R). As per the C-A and T-C-A domain substitutions, the T domain substitution plasmids were transformed into *P. aeruginosa* PAO1.

### Measurement of pyoverdine production

For measuring pyoverdine levels in liquid media, strains were grown in 200 μL of LB in a 96 well plate for 24 h at 37 °C. This starter culture was used to inoculate M9 media containing 0.2 % (w/v) L-arabinose and 4 g/l succinate (pH 7.0) at a 20x dilution and total volume of 200 μL. After 24 h of growth, cultures were centrifuged to pellet bacteria, and then 100 μL of supernatant transferred to a fresh 96 well plate. Supernatant was diluted 2x in fresh M9 media to give a total volume of 200 μL. Fluorescence (ex. 400 nm/ em. 440 nm) and absorbance (400 nm) were measured using an EnSpire 2300 Multilabel Reader (Perkin Elmer, Waltham, MA, USA).

### Mass spectrometry

Cultures were grown according to the methods for measurement of pyoverdine production, above. Cells were pelleted by centrifugation and a sample of supernatant was mixed with matrix solution (500 μL acetonitrile, 500 μL ultrapure water, 1 μL trifluoroacetic acid, 10 μg α-Cyano-4-hydroxycinnamic acid) in a volumetric sample to matrix ratio ranging from 1:5 to 1:20. Aliquots of 1 μl from each sample were spotted in duplicate onto an Opti-TOF® 384 well MALDI plate (Applied Biosystems, Foster City, CA) and allowed to dry at room temperature. Spots were analyzed using a MALDI TOF/TOF 5800 mass spectrometer (Applied Biosystems) in positive ion mode. Each spot was externally calibrated using cal2 calibration mixture (Applied Biosystems). Peaks in spectra were labelled in Data Explorer (Applied Biosystems).

## Availability of supporting data

The data sets supporting the results of this article are included within the article and its additional files (Additional file 1: Tables S1, Additional file 2: Table S2, Additional file 3: Table S3).

## Additional files

Additional file 1:
**Supplementary Table S1.** Proteins used for T domain substitutions in this study and percent identity shared between the substituted region and the corresponding sequence from PvdD. (DOCX 19 kb) Additional file 2:
**Supplementary Table S2.** Oligonucleotide primers used in this study. (DOCX 21 kb) Additional file 3:
**Supplementary Table S3.** Plasmids used in this study. (DOCX 20 kb)
