# Supplementary material for: Portability of the thiolation domain in recombinant pyoverdine non-ribosomal peptide synthetases
Source: BMC Microbiol. 2015 Aug 13;15:162. doi: 10.1186/s12866-015-0496-3 (PMC4535683; doi:10.1186/s12866-015-0496-3)
Supplement: Additional file 1: — Supplementary Table S1. Proteins used for T domain substitutions in this study and percent identity shared between the substituted region and the corresponding sequence from PvdD. (DOCX 19 kb) [file 12866_2015_496_MOESM1_ESM.docx]

Additional file 1: Table S1. Proteins used for T domain substitutions in this study and percent identity shared between the substituted region and the corresponding sequence from *pvdD.*

| Substitution | Protein^1^ | GenBank accession | Identity^2^ | Left boundary^3^ | Right boundary^3^ |
| --- | --- | --- | --- | --- | --- |
| CA-Wt | PvdD | NP_251089 | 100 | TTDAV | LPQPD |
| CA-Ser1 | PvdI | NP_251092 | 54.0 | AASAA | LPRPQ |
| TCA-Ser1 | PvdI | NP_251092 | 54.1 | RRQAG | LPRPQ |
| CA-Ser2 | Pspph1925 | YP_274153 | 52.4 | GQGNA | LPTPD |
| TCA-Ser2 | Pspph1925 | YP_274153 | 53.0 | LQAAY | LPTPD |
| CA-fhOrn | Pflu2544 | YP_002872133.1 | 55.0 | QAPGA | LPRPD |
| TCA-fhOrn | Pflu2544 | YP_002872133.1 | 55.5 | VQQAW | LPRPD |
| T1 | PvdD | NP_251089 | 100 | SQQAY | FAQGL |
| C1 | PvdI | NP_251092 | 56.4 | RRQAG | FAASL |
| C2 | Pspph1925 | YP_274153 | 61.5 | RRQAG | FAQAA |
| C3 | Pp4219 | NP_746336 | 51.3 | AQRPY | FAERV |
| C4 | Pflu2543 | YP_002872132.1 | 56.4 | ARQAY | FAAAC |
| C5 | Pflu2543 | YP_002872132.1 | 56.4 | LRTGH | FVQAL |
| C6 | Pflu2544 | YP_002872133.1 | 60.3 | VQQAW | FVAAL |
| Ct1 | PvdI | NP_251092 | 52.6 | LQQVY | YAELA |
| Ct2 | PvdJ | NP_251090 | 53.9 | LQQVY | FCHGV |
| Ct3 | Pspph1924 | YP_274152 | 57.7 | LQAAY | FCATL |
| Ct4 | Pp4220 | NP_746337 | 52.6 | AQQRY | FSEAV |
| E1 | PvdI | NP_251092 | 39.0 | AGQTH | LARVA |
| E2 | Pspph1924 | YP_274152 | 41.6 | VKQRY | LASVA |
| E3 | Pp4220 | NP_746337 | 44.2 | AGKAY | LAQVA |
| E4 | Pflu2543 | YP_002872132.1 | 44.2 | PQQAF | LARVA |
| Te1 | PvdD | NP_251089 | 61.5 | SQQAY | QAACI |
| Te2 | Pspph1926 | YP_274154 | 45.2 | SQQGH | LADFM |
| Te3 | Pp4219 | NP_746336 | 34.6 | ALREH | LTAVA |
| Te4 | Pflu2544 | YP_002872133.1 | 47.4 | GADAY | QVNCL |

^1^PvdI/J/D are NRPS from *P. aeruginosa* PAO1; Pspph, Pflu and Pp refer to *P. syringae* 1448a*, P. fluorescens* SBW25 and *P. putida* KT2440, respectively, with all gene names and numbering as per the official genome databases, available at www.pseudomonas.com.

^2^Sequence identity based on ClustalW2 multiple sequence alignment of the substituted regions.

^3^Left and right boundary refer to the first and last five residues, respectively, of the substituted region.
