# Supplementary material for: Portability of the thiolation domain in recombinant pyoverdine non-ribosomal peptide synthetases
Source: BMC Microbiol. 2015 Aug 13;15:162. doi: 10.1186/s12866-015-0496-3 (PMC4535683; doi:10.1186/s12866-015-0496-3)
Supplement: Additional file 2: — Supplementary Table S2. Oligonucleotide primers used in this study. (DOCX 21 kb) [file 12866_2015_496_MOESM2_ESM.docx]

Additional file 2: Table S2. Oligonucleotide primers used in this study.

Function

Name Sequence (5′ → 3′; restriction sites underlined)

Primers for pSMC

CATfwd ccccGAATTCcatatgCAAGCACTCATAGAGAAGGTG

CATrev CCCCActagTCAATCCCTGGGCGAACGC

TTeFwd cgcccagggattgTctagagcggccgcgTCGCAGCAGGCCTATCGAGCGC

TTeRev ccccgagctcTCAGCGCCCGGCACGCTCCAGG

Forward primers for pSMA C-A domain substitutions

CA-Wt_CAfwd ggggtctagaACGACGGATGCGGTCTCGACGA

Ser-1_CAfwd CCCTCTAGAGCGGCGAGCGCTGCCCCTGTGCT

Ser-2_CAfwd cccctctagaGGGCAGGGCAATGCTGCG

fhOrn_CAfwd CCCCTCTAGACAGGCCCCAGGCGCG

Reverse primers for C-A and T-C-A domain substitutions

CA-Wt_Rev ccccgcggccgcATCCGGTTGCGGCAACGCCTGC

Ser-1_Rev CCCCGCGGCCGCTTGCGGTCGCGGCAGCGCCTTG

Ser-2_Rev ccccgcggccgCGTCTGGTGTCGGCAGGGC

fhOrn-Rev GCGACGCGGCCGCATCCGGGCGCGGCAGG

Foward primers for T-C-A domain substitutions

TCA-Ser1_Fwd CCCCGCGGCCGCCCGCCGCCAGGCCGGAGAGCCT

TCA-Ser2_Fwd CCCCCGCGGCCGCGTTGCAGGCGGCCTACATCGCT

TCA-fhOrn_Fwd CGGATGCGGCCGCGGTGCAACAGGCCTGGCAG

Forward and reverse primers for T1 domain substitutions

CATTE_Fwd CCCGCGGCCGCCCCACTAGTACGACGGATGCGGTCTCGACGA

C1F CCCCGCGGCCGCCCGCCGCCAGGCCGGAGAGCCT

C1R CCCCGCTAGCCAGGGAAGCGGCGAAGTCTGCC

C2F CCCCGCGGCCGCGTTGCAGGCGGCCTACATCGCT

C2R CCCGCTAGCCGCAGCCTGGGCGAAAGCTGCC

C3F CCCCGCGGCCGCCGCCCAGCGCCCGTACCAGGCG

C3R CCCCGCTAGCCACCCGCTCGGCGAAGCCCGCC

C4F CCCCGCGGCCGCGGCGCGCCAGGCCTAC

C4R CCCCGCTAGCGCAAGCGGCTGCGAACG

C5F CCCCGCGGCCGCGTTGCGCACAGGTCATGTGG

C5R CCCCGCTAGCCAGGGCCTGCACGAAGTCAC

C6F CCCCGCGGCCGCGGTGCAACAGGCCTGGCAG

C6R CCCCGCTAGCCAGTGCGGCGACAAACC

E1F CCCCGCGGCCGCGGCGGGGCAGACGCATGT

E1R CCCCGCTAGCGGCGACTCGCGCCAG

E2F CCCCGCGGCCGCGGTCAAACAGCGTTACACCGC

E2R CCCCGCTAGCTGCCACCGAGGCCAGCTG

E3F CCCCGCGGCCGCGGCCGGCAAGGCTTACGTA

E3R CCCCGCTAGCGGCCACCTGGGCCAAGGC

E4F CCCCGCGGCCGCGCCACAGCAGGCCTTTGTCGCT

E4R CCCCGCTAGCCGCCACCCGCGCCAG

Ct1F CCCCGCGGCCGCGTTGCAGCAGGTCTACGTGGCG

Ct1R CCCCGCTAGCGGCAAGCTCGGCATAGGCTTGC

Ct2F CCCCGCGGCCGCGTTGCAGCAGGTCTACGTGGCG

Ct2R CCCCGCTAGCGACGCCGTGGCAGAACTCACCC

Ct3F CCCCGCGGCCGCGTTGCAGGCGGCCTACATCGCT

Ct3R CCCGCTAGCCAGCGTGGCGCAGAAGTCCGCC

Ct4F CCCCGCGGCCGCCGCTCAGCAGCGCTACCAGGCA

Ct4R CCCCGCTAGCGACGGCCTCGCTGAACGCTCCC

TE1F CAACCGGATGCGGCCGCGTCGCAACAGGCCTATCGAGCGCCC

TE1R CATCCGTCGTGCTAGCCAATCCCTGGGCGAACGC

TE2F CCCCGCGGCCGCGTCACAGCAAGGCCACGTC

TE2R CCCCGCTAGCCATGAAATCGGCCAGCTCACC

TE3F CCCCGCGGCCGCGGCGTTGCGTGAACATGTGGCAC

TE3R CCCCGCTAGCCGCCACGGCGGTCAGCTC

TE4F CCCCGCGGCCGCGGGCGCGGACGCTTATCAGG

TE4R CCCCGCTAGCCAGGCAGTTGACCTGGCCGCGT
