# Supplementary material for: Portability of the thiolation domain in recombinant pyoverdine non-ribosomal peptide synthetases
Source: BMC Microbiol. 2015 Aug 13;15:162. doi: 10.1186/s12866-015-0496-3 (PMC4535683; doi:10.1186/s12866-015-0496-3)
Supplement: Additional file 3: — Supplementary Table S3. Plasmids used in this study. (DOCX 20 kb) [file 12866_2015_496_MOESM3_ESM.docx]

Additional file 3: Table S3. Plasmids used in this study.

Plasmid Short description Source

pSW196 Integration proficient vector; P_BAD_ promoter. (1)

pSMC pSW196 based staging plasmid allowing the (2)

substitution of C-A domains into *pvdD*.

pTCA pSW196 based staging plasmid allowing the This study

substitution of T-C-A domains into the second

module of *pvdD*.

pST1 pSW196 based staging plasmid allowing the This study

substitution of T domains into the second

module of pvdD.

References:

1. **Baynham PJ, Ramsey DM, Gvozdyev BV, Cordonnier EM, Wozniak DJ**. 2006. The *Pseudomonas aeruginosa* Ribbon-Helix-Helix DNA-Binding Protein AlgZ (AmrZ) Controls Twitching Motility and Biogenesis of Type IV Pili. J Bacteriol **188**:132–140.

2. **Calcott MJ, Owen JG, Lamont IL, Ackerley DF**. 2014. Biosynthesis of Novel Pyoverdines by Domain Substitution in a Nonribosomal Peptide Synthetase of *Pseudomonas aeruginosa*. Appl Environ Microbiol **80**:5723–5731.
